# Supplementary material for: SARSPLEX: Multiplex Serological ELISA with a Holistic Approach
Source: Viruses. 2022 Nov 22;14(12):2593. doi: 10.3390/v14122593 (PMC9786076; doi:10.3390/v14122593)
Supplement: Supplementary file 1 [file viruses-14-02593-s001.zip › viruses-2034649-supplementary.pdf]

SARSPLEX supplementary line data for each specimen evaluated in the clinical validation study

Tables S1, S2, and S3 present IgM, IgG, and IgA line data from SARSPLEX validation, respectively. The optical density values (ODI) in red, yellow, or green color represent positive, borderline, or negative immune responses, respectively.

Table S1: List of samples selected for SARSPLEX IgM clinical validation. Deidentified ID, as well as external previous diagnosis, are provided for all sample categories that include SARS-CoV-2 antibody positive (*n* =13) and negative (*n* =52), RT-PCR with disease onset < 19 days (*n* =7), > 19 days (*n* =26), and negative (*n* =2), Human Parvovirus B19 positive (*n* = 2), pre-2019CoV (*n* =64), and summer flu (*n* =36). The total sample size for SARSPLEX IgM validation is *n* = 202. Previous diagnosis refers to reference results from RT-PCR tests manufactured by Autoimmun Diagnostik GmBH and Mobidiag Oy or serology tests developed by Euroimmune, Ortho clinical diagnostics, Mindray, and more. IgM ODI refers to the IgM optical density index from SARSPLEX.

Sample ID	Sample origin	Previous diagnosis	IgM ODI
COP2°	Germany	RT-PCR positive	1.647
COP3°	Germany	RT-PCR positive	1.121
COP4°	Germany	RT-PCR positive	0.867
COP5°	Germany	RT-PCR positive	1.566
COP6°	Germany	RT-PCR positive	3.254
COP7°	Germany	RT-PCR negative	1.653
COP8°	Germany	RT-PCR negative	0.682
A184°	Germany	Pre-2019-CoV	0.572
A185°	Germany	Pre-2019-CoV	0.861
A187°	Germany	Pre-2019-CoV	0.867
A189°	Germany	Pre-2019-CoV	0.572
A193°	Germany	Pre-2019-CoV	0.671
A194°	Germany	Pre-2019-CoV	0.613
A226°	Germany	Pre-2019-CoV	0.919
A237°	Germany	Pre-2019-CoV	0.931
A246°	Germany	Pre-2019-CoV	0.994
A265°	Germany	Pre-2019-CoV	0.838
A267°	Germany	Pre-2019-CoV	0.694
A276°	Germany	Pre-2019-CoV	0.711
A292°	Germany	Pre-2019-CoV	0.555
A293°	Germany	Pre-2019-CoV	0.353
A298°	Germany	Pre-2019-CoV	0.503

A306°	Germany	Pre-2019-CoV	1.029
A308°	Germany	Pre-2019-CoV	0.740
A312°	Germany	Pre-2019-CoV	0.965
A315°	Germany	Pre-2019-CoV	0.671
A316°	Germany	Pre-2019-CoV	0.618
A317°	Germany	Pre-2019-CoV	0.919
A318°	Germany	Pre-2019-CoV	0.462
A321°	Germany	Pre-2019-CoV	0.844
A328°	Germany	Pre-2019-CoV	0.566
A331°	Germany	Pre-2019-CoV	0.844
A334°	Germany	Pre-2019-CoV	0.827
A335°	Germany	Pre-2019-CoV	0.618
A336°	Germany	Pre-2019-CoV	0.775
A341°	Germany	Pre-2019-CoV	0.474
A353°	Germany	Pre-2019-CoV	0.734
A355°	Germany	Pre-2019-CoV	0.775
A363°	Germany	Pre-2019-CoV	0.867
A366°	Germany	Pre-2019-CoV	0.723
A367°	Germany	Pre-2019-CoV	0.624
A375°	Germany	Pre-2019-CoV	0.618
A376°	Germany	Pre-2019-CoV	0.965
UM79*	Finland	Summer flu	0.879
UM81*	Finland	Summer flu	0.578
UM82*	Finland	Summer flu	0.896
UM83*	Finland	Summer flu	0.867
UM84*	Finland	Summer flu	0.844
UM87*	Finland	Summer flu	0.786
UM88*	Finland	Summer flu	0.659
UM92*	Finland	Summer flu	0.815
UM96*	Finland	Summer flu	0.775
UM99*	Finland	Summer flu	0.769
UM100*	Finland	Summer flu	0.879
UM102*	Finland	Summer flu	0.399
UM103*	Finland	Summer flu	0.879
UM106*	Finland	Summer flu	0.624
UM107*	Finland	Summer flu	0.543
UM109*	Finland	Summer flu	0.601
UM111*	Finland	Summer flu	0.971
UM112*	Finland	Summer flu	0.867
UM114*	Finland	Summer flu	0.786
UM115*	Finland	Summer flu	0.538
UM117*	Finland	Summer flu	0.931

UM120*	Finland	Summer flu	0.763
UM121*	Finland	Summer flu	0.613
UM122*	Finland	Summer flu	0.410
UM123*	Finland	Summer flu	0.526
UM124*	Finland	Summer flu	0.844
UM125*	Finland	Summer flu	0.543
UM126*	Finland	Summer flu	0.647
UM131*	Finland	Summer flu	0.624
UM132*	Finland	Summer flu	0.653
UM133*	Finland	Summer flu	0.821
UM134*	Finland	Summer flu	0.642
UM135*	Finland	Summer flu	0.954
UM139*	Finland	Summer flu	0.665
UM141*	Finland	Summer flu	0.555
UM145*	Finland	Summer flu	1.006
274*	Spain	QMLAB NEG & ELISA IgM NEG	0.486
275*	Spain	ELISA IgM POS	1.104
2219*	Spain	QMLAB NEG & ELISA IgM NEG	0.451
2233*	Spain	QMLAB NEG & ELISA IgM NEG	0.717
4604*	Spain	QMLAB POS & ELISA IgM POS	0.792
4612*	Spain	QMLAB NEG & ELISA IgM NEG	0.751
4613*	Spain	QMLAB NEG & ELISA IgM NEG	0.509
4614*	Spain	ELISA IgM NEG	0.434
4623*	Spain	QMLAB NEG & ELISA IgM NEG	0.728
4626*	Spain	ELISA IgM NEG	0.676
4627*	Spain	ELISA IgM NEG	0.445
4630*	Spain	QMLAB POS & ELISA IgM POS	0.873
4631*	Spain	QMLAB POS	1.104
4632*	Spain	ELISA IgM NEG	0.705
4633*	Spain	QMLAB POS & ELISA IgM POS	0.728
4634*	Spain	QMLAB NEG & ELISA IgM NEG	0.486
4635*	Spain	ELISA IgM NEG	0.665
4637*	Spain	QMLAB NEG & ELISA IgM NEG	0.364
4638*	Spain	ELISA IgM NEG	0.705
8243*	Spain	ELISA IgM NEG	0.416
8255*	Spain	QMLAB NEG	0.590
23580*	Spain	QMLAB NEG & ELISA IgM NEG	0.653
23589*	Spain	ELISA IgM NEG	0.751
23590*	Spain	QMLAB POS	1.110
23597*	Spain	QMLAB NEG & ELISA IgM NEG	0.699
10709*	Spain	QMLAB NEG	0.543
66799*	Spain	QMLAB NEG & ELISA IgM NEG	0.355

8219*	Spain	QMLAB NEG & ELISA IgM NEG	0.397
4701*	Spain	QMLAB NEG & ELISA IgM NEG	0.372
4650*	Spain	QMLAB NEG & ELISA IgM NEG	0.502
4642*	Spain	ELISA IgM NEG	0.550
4643*	Spain	QMLAB NEG & ELISA IgM NEG	0.426
4644*	Spain	ELISA IgM NEG	0.835
8190*	Spain	ELISA IgM NEG	0.751
8196*	Spain	QMLAB NEG & ELISA IgM NEG	0.491
8202*	Spain	ELISA IgM NEG	0.509
8203*	Spain	QMLAB POS	1.118
8204*	Spain	ELISA IgM NEG	0.372
8206*	Spain	ELISA IgM NEG	0.308
8209*	Spain	ELISA IgM NEG	0.343
8210*	Spain	ELISA IgM NEG	0.408
8211*	Spain	ELISA IgM NEG	0.544
9064*	Spain	QMLAB NEG	0.337
9065*	Spain	QMLAB NEG	0.443
9076*	Spain	QMLAB NEG & ELISA IgM NEG	0.461
9078*	Spain	QMLAB NEG & ELISA IgM NEG	0.520
9080*	Spain	ELISA IgM NEG	0.486
23548*	Spain	ELISA IgM NEG	0.621
23555*	Spain	QMLAB NEG & ELISA IgM NEG	0.372
23556*	Spain	QMLAB NEG & ELISA IgM NEG	0.313
23565*	Spain	ELISA IgM NEG	0.420
POS1*	Sweden	RT-PCR Positive sample collected 16 days after disease onset	0.589
POS2*	Sweden	RT-PCR Positive sample collected 15 days after disease onset	1.152
POS3*	Sweden	RT-PCR Positive sample collected 16 days after disease onset	0.675
POS4*	Sweden	RT-PCR Positive sample collected 16 days after disease onset	0.506
POS5*	Sweden	RT-PCR Positive sample collected 16 days after disease onset	0.476
POS6*	Sweden	RT-PCR Positive sample collected 15 days after disease onset	0.382
POS7*	Sweden	RT-PCR Positive sample collected 15 days after disease onset	2.624
POS8*	Sweden	RT-PCR Positive sample collected 25 days after disease onset	0.389
POS9*	Sweden	RT-PCR Positive sample collected 22 days after disease onset	4.010

POS10*	Sweden	RT-PCR Positive sample collected 19 days after disease onset	2.401
POS11*	Sweden	RT-PCR Positive sample	2.829
POS12*	Sweden	RT-PCR Positive sample collected 25 days after disease onset	0.479
POS13*	Sweden	RT-PCR Positive sample collected 33 days after disease onset	0.445
POS14*	Sweden	RT-PCR Positive sample collected 30 days after disease onset	0.557
POS15*	Sweden	RT-PCR Positive sample collected 24 days after disease onset	1.203
POS16*	Sweden	RT-PCR Positive sample collected 26 days after disease onset	1.046
POS17*	Sweden	RT-PCR Positive sample collected 32 days after disease onset	6.784
POS18*	Sweden	RT-PCR Positive sample collected 26 days after disease onset	1.250
POS19*	Sweden	RT-PCR Positive sample collected 26 days after disease onset	7.461
POS20*	Sweden	RT-PCR Positive sample	7.342
POS21*	Sweden	RT-PCR Positive sample collected 33 days after disease onset	1.357
POS22*	Sweden	RT-PCR Positive sample	4.991
POS23*	Sweden	RT-PCR Positive sample collected 21 days after disease onset	3.399
POS24*	Sweden	RT-PCR Positive sample collected 22 days after disease onset	1.271
POS25*	Sweden	RT-PCR Positive sample collected 49 days after disease onset	19.670
POS26*	Sweden	RT-PCR Positive sample collected 23 days after disease onset	1.134
POS27*	Sweden	RT-PCR Positive sample collected 58 days after disease onset	1.730
POS28*	Sweden	RT-PCR Positive sample collected 67 days after disease onset	0.719
BRH1433663*	Sweden	Pre-2019-CoV	0.366
BRH1422641*	Sweden	Pre-2019-CoV	0.404
BRH1181285*	Sweden	Pre-2019-CoV	0.431
BRH1181287*	Sweden	Pre-2019-CoV	0.410
BRH1628895*	Sweden	Pre-2019-CoV	0.399
BRH1628903*	Sweden	Pre-2019-CoV	0.545
BRH1628904*	Sweden	Pre-2019-CoV	0.379
BRH1628909*	Sweden	Pre-2019-CoV	0.382

BRH1628916*	Sweden	Pre-2019-CoV	0.415
BRH1628925*	Sweden	Pre-2019-CoV	0.325
BRH1628936*	Sweden	Pre-2019-CoV	0.357
BRH1628941*	Sweden	Pre-2019-CoV	0.512
HMN28941*	Sweden	Pre-2019-CoV	0.557
BRH1422669*	Sweden	Pre-2019-CoV	0.879
BRH1422674*	Sweden	Pre-2019-CoV	0.703
BRH1433675*	Sweden	Pre-2019-CoV	0.811
BRH1433696*	Sweden	Pre-2019-CoV	1.324
BRH1433655*	Sweden	Pre-2019-CoV	0.429
BRH1433656*	Sweden	Pre-2019-CoV	0.615
BRH1433665*	Sweden	Pre-2019-CoV	0.962
BRH1422632*	Sweden	Pre-2019-CoV	6.196
BRH1422642*	Sweden	Pre-2019-CoV	2.399
BRH1422651*	Sweden	Pre-2019-CoV	0.739
BRH1422654*	Sweden	Pre-2019-CoV	0.738
BRH1422656*	Sweden	Pre-2019-CoV	0.987
BRH1422668*	Sweden	Pre-2019-CoV	1.140
Linköping NS1*	Sweden	Pre-2019-CoV	1.094
Linköping NS2*	Sweden	Pre-2019-CoV	1.623
2°	Russia	Mindray IgM POS	1.000
4°	Russia	Mindray IgM POS	2.913
5°	Russia	Mindray IgM POS	1.422
8°	Russia	Mindray IgM POS	1.445
10°	Russia	Mindray IgM POS	1.162
19°	Russia	Mindray IgM POS	1.098
20°	Russia	Mindray IgM NEG	0.647
21°	Russia	Mindray IgM NEG	0.832
22°	Russia	Mindray IgM NEG	0.520
23°	Russia	Mindray IgM NEG	0.780
25°	Russia	Mindray IgM NEG	0.491
26°	Russia	Mindray IgM NEG	0.971
27°	Russia	Mindray IgM NEG	0.832
28°	Russia	Mindray IgM NEG	0.601
DLS0087200 (202)*	Finland	Human Parvovirus B19	0.532
DLS0077499 (203)*	Finland	Human Parvovirus B19	0.717

*Serum was collected using clot activator serum tubes or SST II Advance tubes

°Plasma was collected using a Lithium Heparin tube.

POS = Positive clinical outcome

NEG = Negative clinical outcome

Table S2: List of samples selected for SARSPLEX IgG clinical validation. Deidentified ID, as well as external previous diagnosis, are provided for all sample categories that include SARS-CoV-2 antibody positive (*n* =67) and negative (*n* =78), RT-PCR with disease onset < 19 days (*n* =7), > 19 days (*n* =26), and negative (*n* =2), Human Parvovirus B19 positive (*n* = 2), pre-2019CoV (*n* =102), and summer flu (*n* =44). The total sample size for SARSPLEX IgG validation is *n* = 328. Previous diagnosis refers to reference results from RT-PCR tests manufactured by Autoimmun Diagnostik GmBH and Mobidiag Oy or serology tests developed by Euroimmune, Ortho clinical diagnostics, Mindray, and more. IgG ODI refers to the IgG optical density index from SARSPLEX.

Sample ID	Sample origin	Previous diagnosis	IgG ODI
COP1°	Germany	IgG weak positive	1.204
COP2°	Germany	IgG positive & RTPCR POS	1.164
COP3°	Germany	IgG positive & RTPCR POS	1.788
COP4°	Germany	IgG positive & RTPCR POS	2.617
COP5°	Germany	IgG positive & RTPCR POS	2.920
COP6°	Germany	IgG negative & RTPCR POS	1.407
COP7°	Germany	IgG negative & RTPCR NEG	0.864
COP8°	Germany	IgG negative & RTPCR NEG	0.317
COP9°	Germany	IgG positive	1.556
COP10°	Germany	IgG positive	2.920
COP11°	Germany	IgG negative	1.034
COP12°	Germany	IgG positive	1.738
COP13°	Germany	IgG positive	2.757
COP14°	Germany	IgG negative	0.395
COP15°	Germany	IgG negative	0.622
COP16°	Germany	IgG negative	0.348
COP17°	Germany	IgG positive	0.343
COP115*	Germany	IgG positive	1.061
COP116*	Germany	IgG negative	0.395
COP117*	Germany	IgG negative	0.707
COP118*	Germany	IgG negative	0.291
COP119*	Germany	IgG negative	0.563
COP120*	Germany	IgG negative	0.305
COP121*	Germany	IgG negative	0.385
COP122*	Germany	IgG negative	0.376
COP123*	Germany	IgG negative	0.559
COP124*	Germany	IgG negative	0.879
COP125*	Germany	IgG negative	0.378
COP126*	Germany	IgG negative	0.330
COP127*	Germany	IgG negative	0.596
COP128*	Germany	IgG negative	0.244

COP129*	Germany	IgG negative	0.450
COP130*	Germany	IgG negative	0.347
COP131*	Germany	IgG negative	0.524
COP132*	Germany	IgG negative	0.495
COP133*	Germany	IgG negative	0.170
COP134*	Germany	IgG negative	0.679
COP135*	Germany	IgG negative	0.467
COP136*	Germany	IgG negative	0.184
COP137*	Germany	IgG negative	0.298
COP138*	Germany	IgG negative	0.509
COP139*	Germany	IgG negative	0.316
COP140*	Germany	IgG negative	0.287
COP141*	Germany	IgG negative	0.259
COP142*	Germany	IgG negative	1.129
A4°	Germany	Pre-2019-CoV	0.637
A5°	Germany	Pre-2019-CoV	0.724
A82°	Germany	Pre-2019-CoV	0.555
A83°	Germany	Pre-2019-CoV	0.741
A84°	Germany	Pre-2019-CoV	0.562
A87°	Germany	Pre-2019-CoV	0.814
A91°	Germany	Pre-2019-CoV	0.843
A93°	Germany	Pre-2019-CoV	0.895
A94°	Germany	Pre-2019-CoV	0.402
A97°	Germany	Pre-2019-CoV	0.874
A99°	Germany	Pre-2019-CoV	0.788
A100°	Germany	Pre-2019-CoV	0.655
A103°	Germany	Pre-2019-CoV	0.261
A107°	Germany	Pre-2019-CoV	0.223
A108°	Germany	Pre-2019-CoV	0.207
A112°	Germany	Pre-2019-CoV	1.007
A114°	Germany	Pre-2019-CoV	0.307
A115°	Germany	Pre-2019-CoV	0.496
A116°	Germany	Pre-2019-CoV	0.482
A117°	Germany	Pre-2019-CoV	0.418
A118°	Germany	Pre-2019-CoV	0.571
A119°	Germany	Pre-2019-CoV	0.847
A120°	Germany	Pre-2019-CoV	0.506
A122°	Germany	Pre-2019-CoV	0.323
A123°	Germany	Pre-2019-CoV	0.646
A125°	Germany	Pre-2019-CoV	0.328

A126°	Germany	Pre-2019-CoV	0.414
A129°	Germany	Pre-2019-CoV	0.385
A130°	Germany	Pre-2019-CoV	0.611
A131°	Germany	Pre-2019-CoV	0.490
A133°	Germany	Pre-2019-CoV	0.521
A134°	Germany	Pre-2019-CoV	0.560
A135°	Germany	Pre-2019-CoV	0.324
A154°	Germany	Pre-2019-CoV	0.636
A182°	Germany	Pre-2019-CoV	0.360
A184°	Germany	Pre-2019-CoV	0.568
A185°	Germany	Pre-2019-CoV	0.518
A187°	Germany	Pre-2019-CoV	0.473
A189°	Germany	Pre-2019-CoV	0.307
A192°	Germany	Pre-2019-CoV	0.315
A193°	Germany	Pre-2019-CoV	0.272
A194°	Germany	Pre-2019-CoV	0.625
A226°	Germany	Pre-2019-CoV	0.704
A237°	Germany	Pre-2019-CoV	0.338
A246°	Germany	Pre-2019-CoV	0.642
A252°	Germany	Pre-2019-CoV	0.415
A265°	Germany	Pre-2019-CoV	0.411
A267°	Germany	Pre-2019-CoV	0.534
A276°	Germany	Pre-2019-CoV	0.420
A292°	Germany	Pre-2019-CoV	0.396
A293°	Germany	Pre-2019-CoV	0.697
A298°	Germany	Pre-2019-CoV	0.441
A306°	Germany	Pre-2019-CoV	0.607
A308°	Germany	Pre-2019-CoV	0.318
A312°	Germany	Pre-2019-CoV	0.324
A315°	Germany	Pre-2019-CoV	0.277
A316°	Germany	Pre-2019-CoV	0.542
A318°	Germany	Pre-2019-CoV	0.385
A321°	Germany	Pre-2019-CoV	0.897
A325°	Germany	Pre-2019-CoV	0.447
A328°	Germany	Pre-2019-CoV	0.539
A331°	Germany	Pre-2019-CoV	0.690
A334°	Germany	Pre-2019-CoV	0.478
A335°	Germany	Pre-2019-CoV	0.564
A336°	Germany	Pre-2019-CoV	0.774
A341°	Germany	Pre-2019-CoV	0.236

A344°	Germany	Pre-2019-CoV	0.401
A353°	Germany	Pre-2019-CoV	0.091
A355°	Germany	Pre-2019-CoV	0.350
A363°	Germany	Pre-2019-CoV	0.236
A366°	Germany	Pre-2019-CoV	0.614
A367°	Germany	Pre-2019-CoV	0.509
A375°	Germany	Pre-2019-CoV	0.358
A376°	Germany	Pre-2019-CoV	0.267
UM79*	Finland	Summer flu	0.469
UM81*	Finland	Summer flu	0.322
UM82*	Finland	Summer flu	0.634
UM83*	Finland	Summer flu	0.614
UM84*	Finland	Summer flu	0.430
UM86*	Finland	Summer flu	0.393
UM87*	Finland	Summer flu	0.525
UM88*	Finland	Summer flu	0.552
UM92*	Finland	Summer flu	0.626
UM93*	Finland	Summer flu	0.647
UM95*	Finland	Summer flu	0.525
UM96*	Finland	Summer flu	0.871
UM97*	Finland	Summer flu	0.696
UM100*	Finland	Summer flu	0.713
UM101*	Finland	Summer flu	0.966
UM102*	Finland	Summer flu	0.520
UM103*	Finland	Summer flu	0.446
UM104*	Finland	Summer flu	0.318
UM105*	Finland	Summer flu	0.668
UM106*	Finland	Summer flu	0.687
UM107*	Finland	Summer flu	0.622
UM108*	Finland	Summer flu	0.591
UM109*	Finland	Summer flu	0.213
UM111*	Finland	Summer flu	0.643
UM112*	Finland	Summer flu	0.709
UM114*	Finland	Summer flu	0.409
UM115*	Finland	Summer flu	0.647
UM117*	Finland	Summer flu	1.018
UM121*	Finland	Summer flu	0.623
UM122*	Finland	Summer flu	0.519
UM123*	Finland	Summer flu	0.569
UM124*	Finland	Summer flu	0.269

UM125*	Finland	Summer flu	0.253
UM126*	Finland	Summer flu	0.645
UM128*	Finland	Summer flu	0.467
UM131*	Finland	Summer flu	0.764
UM132*	Finland	Summer flu	0.278
UM133*	Finland	Summer flu	0.434
UM134*	Finland	Summer flu	0.277
UM135*	Finland	Summer flu	0.641
UM138*	Finland	Summer flu	0.380
UM139*	Finland	Summer flu	0.813
UM141*	Finland	Summer flu	0.805
UM145*	Finland	Summer flu	0.253
POS1*	Sweden	RT-PCR Positive sample collected 16 days after disease onset and ELISA IgG NEG	0.316
POS2*	Sweden	RT-PCR Positive sample collected 15 days after disease onset and ELISA IgG NEG	0.676
POS3*	Sweden	RT-PCR Positive sample collected 16 days after disease onset and ELISA IgG NEG	0.929
POS4*	Sweden	RT-PCR Positive sample collected 16 days after disease onset and ELISA IgG NEG	0.949
POS5*	Sweden	RT-PCR Positive sample collected 16 days after disease onset and ELISA IgG NEG	0.348
POS6*	Sweden	RT-PCR Positive sample collected 15 days after disease onset and ELISA IgG NEG	0.652
POS7*	Sweden	RT-PCR Positive sample collected 15 days after disease onset and ELISA IgG NEG	0.308
POS8*	Sweden	RT-PCR Positive sample collected 25 days after disease onset and ELISA IgG POS	0.246
POS9*	Sweden	RT-PCR Positive sample collected 22 days after disease onset and ELISA IgG POS	2.920

POS10*	Sweden	RT-PCR Positive sample collected 19 days after disease onset and ELISA IgG POS	0.156
POS11*	Sweden	RT-PCR Positive sample and ELISA IgG POS	0.239
POS12*	Sweden	RT-PCR Positive sample collected 25 days after disease onset and ELISA IgG POS	1.931
POS13*	Sweden	RT-PCR Positive sample collected 33 days after disease onset and ELISA IgG NEG	0.723
POS14*	Sweden	RT-PCR Positive sample collected 30 days after disease onset and ELISA IgG NEG	0.675
POS15*	Sweden	RT-PCR Positive sample collected 24 days after disease onset and ELISA IgG NEG	1.212
POS16*	Sweden	RT-PCR Positive sample collected 26 days after disease onset and ELISA IgG NEG	0.445
POS17*	Sweden	RT-PCR Positive sample collected 32 days after disease onset and ELISA IgG POS	2.920
POS18*	Sweden	RT-PCR Positive sample collected 26 days after disease onset and ELISA IgG NEG	0.710
POS19*	Sweden	RT-PCR Positive sample collected 26 days after disease onset and ELISA IgG POS	2.920
POS20*	Sweden	RT-PCR Positive sample and ELISA IgG POS	2.920
POS21*	Sweden	RT-PCR Positive sample collected 33 days after disease onset and ELISA IgG POS	2.920
POS22*	Sweden	RT-PCR Positive sample and ELISA IgG POS	2.920
POS23*	Sweden	RT-PCR Positive sample collected 21 days after disease onset and ELISA IgG POS	2.411

POS24*	Sweden	RT-PCR Positive sample collected 22 days after disease onset and ELISA IgG POS	2.920
POS25*	Sweden	RT-PCR Positive sample collected 49 days after disease onset and ELISA IgG POS	2.920
POS26*	Sweden	RT-PCR Positive sample collected 23 days after disease onset and ELISA IgG POS	2.746
POS27*	Sweden	RT-PCR Positive sample collected 58 days after disease onset and ELISA IgG POS	2.792
POS28*	Sweden	RT-PCR Positive sample collected 67 days after disease onset and ELISA IgG POS	2.566
BRH1433663*	Sweden	Pre-2019-CoV	0.351
BRH1422641*	Sweden	Pre-2019-CoV	0.761
BRH1181285*	Sweden	Pre-2019-CoV	0.232
BRH1181287*	Sweden	Pre-2019-CoV	0.244
BRH1628895*	Sweden	Pre-2019-CoV	0.272
BRH1628903*	Sweden	Pre-2019-CoV	0.291
BRH1628904*	Sweden	Pre-2019-CoV	2.104
BRH1628909*	Sweden	Pre-2019-CoV	1.552
BRH1628916*	Sweden	Pre-2019-CoV	1.264
BRH1628925*	Sweden	Pre-2019-CoV	0.399
BRH1628936*	Sweden	Pre-2019-CoV	0.395
BRH1628941*	Sweden	Pre-2019-CoV	0.301
HMN28941*	Sweden	Pre-2019-CoV	0.308
BRH1422669*	Sweden	Pre-2019-CoV	0.251
BRH1422674*	Sweden	Pre-2019-CoV	0.583
BRH1433675*	Sweden	Pre-2019-CoV	0.537
BRH1433696*	Sweden	Pre-2019-CoV	1.508
BRH1433655*	Sweden	Pre-2019-CoV	0.742
BRH1433656*	Sweden	Pre-2019-CoV	0.696
BRH1433665*	Sweden	Pre-2019-CoV	1.405
BRH1422632*	Sweden	Pre-2019-CoV	1.527
BRH1422642*	Sweden	Pre-2019-CoV	0.800
BRH1422651*	Sweden	Pre-2019-CoV	1.243
BRH1422654*	Sweden	Pre-2019-CoV	1.829
BRH1422656*	Sweden	Pre-2019-CoV	0.854
BRH1422668*	Sweden	Pre-2019-CoV	0.739

Linköping NS1*	Sweden	Pre-2019-CoV	0.897
Linköping NS2*	Sweden	Pre-2019-CoV	1.895
271*	Spain	QMLAB POS & QML IgG POS	3.104
274*	Spain	QMLAB NEG & QML IgG NEG	0.796
275*	Spain	QMLAB NEG & QML IgG NEG	0.737
2219*	Spain	QMLAB NEG & QML IgG NEG	0.514
4604*	Spain	QMLAB POS & QML IgG POS	3.346
4612*	Spain	QMLAB NEG & QML IgG NEG	0.591
4613*	Spain	QMLAB NEG & QML IgG NEG	0.749
4614*	Spain	QMLAB POS & QML IgG POS	2.708
4623*	Spain	QMLAB NEG & QML IgG NEG	0.266
4626*	Spain	QMLAB POS & QML IgG POS	2.016
4627*	Spain	QMLAB POS & QML IgG POS	2.336
4631*	Spain	QMLAB POS & QML IgG POS	3.196
4632*	Spain	QMLAB POS & QML IgG POS	2.431
4633*	Spain	QMLAB POS & QML IgG POS	3.181
4634*	Spain	QMLAB NEG & QML IgG NEG	0.429
4635*	Spain	QMLAB POS & QML IgG POS	2.859
4637*	Spain	QMLAB NEG & QML IgG NEG	0.610
4638*	Spain	QMLAB POS & QML IgG POS	0.539
8243*	Spain	QMLAB NEG & QML IgG NEG	0.755
8255*	Spain	QMLAB NEG & QML IgG NEG	0.515
23580*	Spain	QMLAB NEG & QML IgG NEG	0.305
10709*	Spain	QMLAB NEG & QML IgG NEG	0.525
66799*	Spain	QMLAB NEG & QML IgG NEG	0.801
8219*	Spain	QMLAB NEG & QML IgG NEG	1.054
4701*	Spain	QMLAB NEG & QML IgG NEG	0.487
4650*	Spain	QMLAB NEG & QML IgG NEG	0.653
4643*	Spain	QMLAB NEG	0.180
4644*	Spain	QMLAB POS & QML IgG POS	1.273
8196*	Spain	QMLAB NEG	0.335
8202*	Spain	QMLAB POS & QML IgG POS	2.717
8203*	Spain	QMLAB POS & QML IgG POS	2.306
8204*	Spain	QML IgG NEG	0.517
8205*	Spain	QMLAB POS & QML IgG POS	2.805
8206*	Spain	QMLAB POS & QML IgG POS	2.706
8209*	Spain	QMLAB NEG & QML IgG NEG	0.661
8210*	Spain	QMLAB NEG & QML IgG NEG	0.451
8211*	Spain	QMLAB POS & QML IgG POS	2.351
9063*	Spain	QMLAB POS & QML IgG POS	1.037

9064*	Spain	QMLAB NEG & QML IgG NEG	0.344
9065*	Spain	QMLAB NEG & QML IgG NEG	0.291
9076*	Spain	QMLAB NEG & QML IgG NEG	0.343
9078*	Spain	QMLAB NEG & QML IgG NEG	0.243
9080*	Spain	QMLAB POS	1.860
23548*	Spain	QMLAB POS	1.001
23555*	Spain	QMLAB NEG & QML IgG NEG	0.292
23556*	Spain	QMLAB NEG & QML IgG NEG	0.395
23565*	Spain	QMLAB POS	1.000
1°	Russia	Mindray IgG POS	2.555
2°	Russia	Mindray IgG POS	2.190
3°	Russia	Mindray IgG POS	2.555
4°	Russia	Mindray IgG POS	2.555
5°	Russia	Mindray IgG POS	2.555
6°	Russia	Mindray IgG POS	2.555
7°	Russia	Mindray IgG POS	2.190
8°	Russia	Mindray IgG POS	2.482
9°	Russia	Mindray IgG POS	2.190
10°	Russia	Mindray IgG POS	2.628
12°	Russia	Mindray IgG NEG	0.971
13°	Russia	Mindray IgG NEG	0.382
14°	Russia	Mindray IgG NEG	0.534
16°	Russia	Mindray IgG NEG	0.909
18°	Russia	Mindray IgG NEG	0.682
19°	Russia	Mindray IgG NEG	1.818
20°	Russia	Mindray IgG POS	2.482
21°	Russia	Mindray IgG POS	1.297
22°	Russia	Mindray IgG POS	1.700
23°	Russia	Mindray IgG POS	2.426
24°	Russia	Mindray IgG POS	2.115
25°	Russia	Mindray IgG POS	1.977
26°	Russia	Mindray IgG POS	2.555
27°	Russia	Mindray IgG POS	2.219
28°	Russia	Mindray IgG POS	1.101
DLS0087200 (202)*	Finland	Human Parvovirus B19	0.980
DLS0077499 (203)*	Finland	Human Parvovirus B19	2.920

*Serum was collected using clot activator serum tubes or SST II Advance tubes

°Plasma was collected using a Lithium Heparin tube.

POS = Positive clinical outcome

NEG = Negative clinical outcome

Table S3: List of samples selected for SARSPLEX IgA clinical validation. Deidentified ID, as well as external previous diagnosis, are provided for all sample categories that include SARS-CoV-2 antibody positive (*n* =23) and negative (*n* =22), RT-PCR with disease onset < 19 days (*n* =7), > 19 days (*n* =26), and negative (*n* =2), Human Parvovirus B19 positive (*n* = 2), pre-2019CoV (*n* =39), and summer flu (*n* =25). The total sample size for SARSPLEX IgA validation is *n* = 146. Previous diagnosis refers to reference results from RT-PCR tests manufactured by Autoimmun Diagnostik GmBH and Mobidiag Oy or serology tests developed by Euroimmune, Ortho clinical diagnostics, Mindray and more. IgA ODI refers to the IgA optical density index from SARSPLEX.

Sample ID	Sample origin	Previous diagnosis	IgA ODI
COP1°	Germany	IgA positive	0.585
COP2°	Germany	IgA positive & RTPCR POS	1.799
COP3°	Germany	IgA positive & RTPCR POS	1.095
COP4°	Germany	IgA positive & RTPCR POS	7.843
COP5°	Germany	IgA positive & RTPCR POS	1.495
COP6°	Germany	IgA positive & RTPCR POS	1.242
COP7°	Germany	IgA positive & RTPCR NEG	1.085
COP8°	Germany	IgA negative & RTPCR NEG	0.544
COP9°	Germany	IgA positive	0.665
COP10°	Germany	IgA positive	5.747
COP11°	Germany	IgA positive	10.294
COP12°	Germany	IgA positive	1.376
COP13°	Germany	IgA positive	8.438
COP14°	Germany	IgA positive	1.119
COP15°	Germany	IgA positive	0.928
COP16°	Germany	IgA negative	0.567
COP17°	Germany	IgA positive	0.881
COP115*	Germany	IgA positive	1.359
COP116*	Germany	IgA positive	0.628
COP117*	Germany	IgA negative	0.572
COP118*	Germany	IgA negative	0.422
COP119*	Germany	IgA negative	0.642
COP120*	Germany	IgA negative	0.553
COP121*	Germany	IgA negative	0.408
COP122*	Germany	IgA positive	0.619
COP123*	Germany	IgA negative	0.427
COP124*	Germany	IgA negative	2.283
COP125*	Germany	IgA negative	0.394
COP126*	Germany	IgA negative	0.633
COP127*	Germany	IgA positive	0.741
COP128*	Germany	IgA negative	0.708
COP129*	Germany	IgA negative	0.666

COP130*	Germany	IgA negative	0.558
COP131*	Germany	IgA negative	0.464
COP132*	Germany	IgA negative	0.563
COP133*	Germany	IgA negative	0.572
COP134*	Germany	IgA positive	0.647
COP135*	Germany	IgA negative	0.558
COP136*	Germany	IgA negative	0.600
COP137*	Germany	IgA negative	0.497
COP138*	Germany	IgA borderline	1.777
COP139*	Germany	IgA negative	0.774
COP140*	Germany	IgA negative	0.403
COP141*	Germany	IgA positive	0.999
COP142*	Germany	IgA positive	4.613
A4°	Germany	Pre-2019-CoV	0.951
A84°	Germany	Pre-2019-CoV	0.856
A91°	Germany	Pre-2019-CoV	0.607
A94°	Germany	Pre-2019-CoV	0.146
A103°	Germany	Pre-2019-CoV	0.739
A107°	Germany	Pre-2019-CoV	0.875
A112°	Germany	Pre-2019-CoV	0.939
A116°	Germany	Pre-2019-CoV	0.819
A117°	Germany	Pre-2019-CoV	0.658
A129°	Germany	Pre-2019-CoV	0.870
A187°	Germany	Pre-2019-CoV	0.707
A192°	Germany	Pre-2019-CoV	0.656
A193°	Germany	Pre-2019-CoV	0.934
A265°	Germany	Pre-2019-CoV	0.719
A276°	Germany	Pre-2019-CoV	0.868
A292°	Germany	Pre-2019-CoV	0.702
A298°	Germany	Pre-2019-CoV	0.685
A335°	Germany	Pre-2019-CoV	0.956
A341°	Germany	Pre-2019-CoV	0.402
A355°	Germany	Pre-2019-CoV	0.422
A363°	Germany	Pre-2019-CoV	0.451
A376°	Germany	Pre-2019-CoV	0.639
UM79*	Finland	Summer flu	0.753
UM82*	Finland	Summer flu	0.778
UM83*	Finland	Summer flu	0.968
UM84*	Finland	Summer flu	0.585
UM86*	Finland	Summer flu	0.653
UM93*	Finland	Summer flu	0.797
UM99*	Finland	Summer flu	0.909

UM103*	Finland	Summer flu	0.895
UM106*	Finland	Summer flu	0.885
UM107*	Finland	Summer flu	0.580
UM114*	Finland	Summer flu	0.861
UM115*	Finland	Summer flu	0.914
UM120*	Finland	Summer flu	0.688
UM121*	Finland	Summer flu	0.661
UM122*	Finland	Summer flu	0.549
UM124*	Finland	Summer flu	0.332
UM125*	Finland	Summer flu	0.414
UM126*	Finland	Summer flu	0.697
UM130*	Finland	Summer flu	0.878
UM132*	Finland	Summer flu	0.763
UM133*	Finland	Summer flu	0.663
UM134*	Finland	Summer flu	0.814
UM135*	Finland	Summer flu	0.746
UM139*	Finland	Summer flu	0.770
UM145*	Finland	Summer flu	0.690
POS1*	Sweden	RT-PCR Positive sample collected 16 days after disease onset	0.764
POS2*	Sweden	RT-PCR Positive sample collected 15 days after disease onset	0.870
POS3*	Sweden	RT-PCR Positive sample collected 16 days after disease onset	3.043
POS4*	Sweden	RT-PCR Positive sample collected 16 days after disease onset	0.678
POS5*	Sweden	RT-PCR Positive sample collected 16 days after disease onset	0.540
POS6*	Sweden	RT-PCR Positive sample collected 15 days after disease onset	0.541
POS7*	Sweden	RT-PCR Positive sample collected 15 days after disease onset	0.754
POS8*	Sweden	RT-PCR Positive sample collected 25 days after disease onset	0.504
POS9*	Sweden	RT-PCR Positive sample collected 22 days after disease onset	3.501
POS10*	Sweden	RT-PCR Positive sample collected 19 days after disease onset	1.824
POS11*	Sweden	RT-PCR Positive sample	0.695
POS12*	Sweden	RT-PCR Positive sample collected 25 days after disease onset	0.577

POS13*	Sweden	RT-PCR Positive sample collected 33 days after disease onset	5.003
POS14*	Sweden	RT-PCR Positive sample collected 30 days after disease onset	0.737
POS15*	Sweden	RT-PCR Positive sample collected 24 days after disease onset	1.007
POS16*	Sweden	RT-PCR Positive sample collected 26 days after disease onset	0.830
POS17*	Sweden	RT-PCR Positive sample collected 32 days after disease onset	20.619
POS18*	Sweden	RT-PCR Positive sample collected 26 days after disease onset	1.114
POS19*	Sweden	RT-PCR Positive sample collected 26 days after disease onset	8.903
POS20*	Sweden	RT-PCR Positive sample	20.619
POS21*	Sweden	RT-PCR Positive sample collected 33 days after disease onset	2.380
POS22*	Sweden	RT-PCR Positive sample	4.158
POS23*	Sweden	RT-PCR Positive sample collected 21 days after disease onset	3.536
POS24*	Sweden	RT-PCR Positive sample collected 22 days after disease onset	2.639
POS25*	Sweden	RT-PCR Positive sample collected 49 days after disease onset	6.770
POS26*	Sweden	RT-PCR Positive sample collected 23 days after disease onset	1.588
POS27*	Sweden	RT-PCR Positive sample collected 58 days after disease onset	5.002
POS28*	Sweden	RT-PCR Positive sample collected 67 days after disease onset	1.793
BRH1433663*	Sweden	Pre-2019-CoV	0.705
BRH1422641*	Sweden	Pre-2019-CoV	0.990
BRH1181285*	Sweden	Pre-2019-CoV	0.527
BRH1181287*	Sweden	Pre-2019-CoV	0.529
BRH1628895*	Sweden	Pre-2019-CoV	0.508
BRH1628903*	Sweden	Pre-2019-CoV	0.522
BRH1628916*	Sweden	Pre-2019-CoV	0.746
BRH1628925*	Sweden	Pre-2019-CoV	0.539
BRH1628936*	Sweden	Pre-2019-CoV	0.497
BRH1628941*	Sweden	Pre-2019-CoV	0.455
HMN28941*	Sweden	Pre-2019-CoV	0.558
BRH1422669*	Sweden	Pre-2019-CoV	0.516

BRH1422674*	Sweden	Pre-2019-CoV	0.858
BRH1433675*	Sweden	Pre-2019-CoV	1.053
BRH1433656*	Sweden	Pre-2019-CoV	1.466
BRH1433665*	Sweden	Pre-2019-CoV	1.074
Linköping NS1*	Sweden	Pre-2019-CoV	1.246
DLS0087200 (202)*	Finland	Human Parvovirus B19	8.351
DLS0077499 (203)*	Finland	Human Parvovirus B19	8.351

*Serum was collected using clot activator serum tubes or SST II Advance tubes

°Plasma was collected using Lithium Heparin tube.

POS = Positive clinical outcome

NEG = Negative clinical outcome

Table S4: Identification of 23 asymptomatic COVID-19 RT-PCR-positive Sanoviv Medical Institute employees. Green negative, red positive, yellow borderline. Diagnosis compared to RT-PCR results.

ID	Sample origin	SARSPLEX IgM optical density index (ODI)	DIAGNOSIS
SMI2	Sanoviv	0,246	RT-PCR Positive
SMI15	Sanoviv	1,22	RT-PCR Positive
SMI18	Sanoviv	0,793	RT-PCR Positive
SMI27	Sanoviv	1,839	RT-PCR Positive
SMI33	Sanoviv	2,405	RT-PCR Positive
SMI39	Sanoviv	0,902	RT-PCR Positive
SMI41	Sanoviv	0,738	RT-PCR Positive
SMI48	Sanoviv	0,908	RT-PCR Positive
SMI51	Sanoviv	0,688	RT-PCR Positive
SMI63	Sanoviv	0,892	RT-PCR Positive
SMI84	Sanoviv	0,929	RT-PCR Positive
SMI89	Sanoviv	0,919	RT-PCR Positive
SMI102	Sanoviv	0,918	RT-PCR Positive
SMI110	Sanoviv	1,882	RT-PCR Negative
SMI110	Sanoviv	0,559	RT-PCR Positive
SMI114	Sanoviv	0,892	RT-PCR Positive

ID	Sample origin	SARSPLEX IgG optical density index (ODI)	DIAGNOSIS
SMI2	Sanoviv	1,209	RT-PCR Positive
SMI15	Sanoviv	1,115	RT-PCR Positive
SMI18	Sanoviv	0,527	RT-PCR Positive
SMI27	Sanoviv	0,593	RT-PCR Positive
SMI33	Sanoviv	0,682	RT-PCR Positive
SMI39	Sanoviv	0,428	RT-PCR Positive
SMI41	Sanoviv	0,691	RT-PCR Positive
SMI48	Sanoviv	0,065	RT-PCR Positive
SMI51	Sanoviv	0,748	RT-PCR Positive
SMI63	Sanoviv	0,692	RT-PCR Positive
SMI84	Sanoviv	1,138	RT-PCR Positive
SMI89	Sanoviv	0,662	RT-PCR Positive
SMI102	Sanoviv	0,792	RT-PCR Positive
SMI110	Sanoviv	1,502	RT-PCR Negative
SMI110	Sanoviv	2,393	RT-PCR Positive
SMI114	Sanoviv	0,682	RT-PCR Positive

ID	Sample origin	SARSPLEX IgA optical density index (ODI)	DIAGNOSIS
SMI2	Sanoviv	1,109	RT-PCR Positive
SMI15	Sanoviv	1,102	RT-PCR Positive
SMI18	Sanoviv	1,682	RT-PCR Positive
SMI27	Sanoviv	2,485	RT-PCR Positive
SMI33	Sanoviv	1,862	RT-PCR Positive
SMI39	Sanoviv	2,637	RT-PCR Positive
SMI41	Sanoviv	1,206	RT-PCR Positive
SMI48	Sanoviv	0,955	RT-PCR Positive
SMI51	Sanoviv	1,834	RT-PCR Positive
SMI63	Sanoviv	1,957	RT-PCR Positive
SMI84	Sanoviv	1,395	RT-PCR Positive
SMI89	Sanoviv	1,763	RT-PCR Positive
SMI102	Sanoviv	2,379	RT-PCR Positive
SMI110	Sanoviv	3,204	RT-PCR Negative
SMI110	Sanoviv	1,844	RT-PCR Positive
SMI114	Sanoviv	2,379	RT-PCR Positive
